# Pericardial Injection of Kainic Acid Induces a Chronic Epileptic State in Larval Zebrafish

**DOI:** 10.3389/fnmol.2021.753936

**Published:** 2021-10-14

**Authors:** Lise Heylen, Duc-Hung Pham, Ann-Sofie De Meulemeester, Éric Samarut, Adrianna Skiba, Daniëlle Copmans, Youcef Kazwiny, Pieter Vanden Berghe, Peter A. M. de Witte, Aleksandra Siekierska

**Affiliations:** ^1^Laboratory for Molecular Biodiscovery, KU Leuven, Leuven, Belgium; ^2^Department of Neurosciences, Research Center of the University of Montreal Hospital Center, University of Montreal, Montreal, QC, Canada; ^3^Modelis Inc., Montreal, QC, Canada; ^4^Laboratory for Enteric Neuroscience, Translational Research Center for Gastrointestinal Disorders, KU Leuven, Leuven, Belgium

**Keywords:** kainic acid, seizures, epilepsy, zebrafish, epileptogenesis, GABA, neuroinflammation

## Abstract

Epilepsy is a common disorder of the brain characterized by spontaneous recurrent seizures, which develop gradually during a process called epileptogenesis. The mechanistic processes underlying the changes of brain tissue and networks toward increased seizure susceptibility are not fully understood. In rodents, injection of kainic acid (KA) ultimately leads to the development of spontaneous epileptic seizures, reflecting similar neuropathological characteristics as seen in patients with temporal lobe epilepsy (TLE). Although this model has significantly contributed to increased knowledge of epileptogenesis, it is technically demanding, costly to operate and hence not suitable for high-throughput screening of anti-epileptic drugs (AEDs). Zebrafish, a vertebrate with complementary advantages to rodents, is an established animal model for epilepsy research. Here, we generated a novel KA-induced epilepsy model in zebrafish larvae that we functionally and pharmacologically validated. KA was administered by pericardial injection at an early zebrafish larval stage. The epileptic phenotype induced was examined by quantification of seizure-like behavior using automated video recording, and of epileptiform brain activity measured via local field potential (LFP) recordings. We also assessed GFP-labeled GABAergic and RFP-labeled glutamatergic neurons in double transgenic KA-injected zebrafish larvae, and examined the GABA and glutamate levels in the larval heads by liquid chromatography with tandem mass spectrometry detection (LC-MS/MS). Finally, KA-injected larvae were exposed to five commonly used AEDs by immersion for pharmacological characterization of the model. Shortly after injection, KA induced a massive damage and inflammation in the zebrafish brain and seizure-like locomotor behavior. An abnormal reorganization of brain circuits was observed, a decrease in both GABAergic and glutamatergic neuronal population and their associated neurotransmitters. Importantly, these changes were accompanied by spontaneous and continuous epileptiform brain discharges starting after a short latency period, as seen in KA rodent models and reminiscent of human pathology. Three out of five AEDs tested rescued LFP abnormalities but did not affect the seizure-like behavior. Taken together, for the first time we describe a chemically-induced larval zebrafish epilepsy model offering unique insights into studying epileptogenic processes *in vivo* and suitable for high-throughput AED screening purposes and rapid genetic investigations.

## Introduction

Epilepsy is a group of neurological diseases characterized by spontaneous recurrent seizures (SRS), resulting from imbalances of excitatory and inhibitory neurotransmission activities in the brain. At the origin of these diseases lies epileptogenesis, a process activated by genetic or acquired factors that is marked by alterations in neuronal excitability and interconnections, most probably as a consequence of structural changes, including cell loss and synaptic reorganization (Pitkanen and Engel, 2014). Evidently, there is an outstanding need to understand the mechanistic processes underlying the changes of brain tissue and networks toward increased seizure susceptibility (Pitkanen et al., 2015).

One of the experimental models that is instrumental to study the mechanistic aspects of epileptogenesis is the kainic acid (KA) rodent model of temporal lobe epilepsy (TLE), which is the most common form of focal epilepsy in adults (Gonzalez Otarula and Schuele, 2020). Administration of KA has long been known to induce behavioral and electrophysiological seizures and neuropathological lesions reminiscent of those occurring in patients with TLE (Levesque and Avoli, 2013). A variety of KA rodent models, comprising different administration routes (Chen et al., 2002; Welzel et al., 2019; Crans et al., 2020) and animal ages (Chen et al., 2002; Berger et al., 2019; Wang et al., 2019; Welzel et al., 2019; Crans et al., 2020) in both rat and mouse, have been widely implemented for varying research purposes (Wang et al., 2019; Welzel et al., 2019; Crans et al., 2020). Even though KA rodent models have substantially increased our understanding of the pathogenesis of SRS, they are technically demanding and costly to operate, and hence not suitable for high-throughput screening of anti-epileptic drugs (AEDs).

Zebrafish, a vertebrate with complementary advantages to rodents, is an established animal model for epilepsy research (Copmans et al., 2017; Yaksi et al., 2021). Moreover, zebrafish platforms enabling medium- and high-throughput screening are increasingly used for drug discovery purposes (Kokel et al., 2010; Baxendale et al., 2012; Dinday and Baraban, 2015; Copmans et al., 2018). The development of morphant and mutant zebrafish models in recent translational research has allowed rapid functional determination of candidate epilepsy genes, such as *stx1b* (Schubert et al., 2014), *prickle1* (Bassuk et al., 2008), *vars* (Siekierska et al., 2019), *scn1lab* (Baraban et al., 2013), and *gabra1* (Samarut et al., 2018). Also proconvulsants, including pentylenetetrazole (Baraban et al., 2005), allylglycine (Leclercq et al., 2015) and ethyl ketopentoate (Zhang et al., 2017) have been used to induce acute epileptic seizures in zebrafish larvae. In comparison with the wide variety of genetic epilepsy models and chemically-induced acute seizure models, the use of chemicals to induce spontaneous recurrent seizures (i.e., chronic epilepsy) in zebrafish larvae has not been reported so far. Previous attempts to incubate zebrafish larvae and adults in KA solution did not result in specific seizure features, but showed different locomotor responses depending on the age of the animal (Menezes et al., 2014). In contrast, perfusion of KA directly through artificial cerebrospinal fluid resulted in local electrographic brain discharges (Kim et al., 2010). Likewise, adult zebrafish injected intraperitoneally with KA showed clonus-like convulsions (Alfaro et al., 2011). These data suggest that KA is not absorbed efficiently by the larvae when dissolved in tank water, probably as a result of its high hydrophilicity. Conversely, pericardial injections represent an easy, efficient and rapid method to deliver substances into the bloodstream of zebrafish larvae allowing systemic diffusion and avoiding brain lesions caused by direct delivery into the brain.

Here we describe a novel KA-induced zebrafish epilepsy model by intrapericardial injection of KA in larvae at three days post fertilization (dpf). We show that injected larvae display whole brain abnormalities followed by spontaneous seizure-like locomotor behavior shortly after KA injection and epileptiform brain discharges after a latency phase, likely due to an altered balance between glutamatergic excitation and GABAergic inhibition. A decrease in epileptiform discharges, but not in seizure-like behavior was observed after treatment with commonly used AEDs. This model provides useful insights into the mechanisms of seizures and epileptogenic processes and could possibly be applicable in the future for the discovery of novel therapeutics including disease-modifying strategies in the fight against drug-resistant epilepsies.

## Materials and Methods

### Zebrafish Husbandry

For all the experiments zebrafish (*Danio rerio*, wild type) lines with *nacre* background were used (Lister et al., 1999). Adult zebrafish of *nacre* and *Tg(dlx5a/dlx6a-EGFP)* (Noble et al., 2015) x *Tg(vglut2a:loxP-RFP-loxP-GFP)* (Satou et al., 2012) strains were maintained under standard aquaculture conditions at 28.5°C on a 14 h light/10 h dark cycle. Fertilized eggs were collected via natural spawning. Embryos and larvae of 0–7 dpf were maintained in Danieau's medium [1.5 mM HEPES, pH 7.6, 17.4 mM NaCl, 0.21 mM KCl, 0.12 mM MgSO_4_, and 0.18 mM Ca(NO_3_)_2_] under the same conditions as adults. All experiments performed at the University of Leuven were approved by the Ethics Committee of the University of Leuven (P061/2013, P023/2017 and P027/2019) and by the Belgian Federal Department of Public Health, Food Safety and Environment (LA1210199).

### Chemicals

A 10 mM stock solution of KA (Sigma) was made in phosphate buffer (PB) and stored at −80°C, three h after preparation thereof. Prior to making final dilutions for injection, a vial containing KA stock solution was thawed and kept at room temperature (RT) for three h. Five percentage dextran rhodamine B (Sigma) was used as an injection marker. As vehicle (VHC), 5% dextran rhodamine B in PB was used. Valproic acid (VPA), levetiracetam (LEV), tiagabine (TGB) and carbamazepine (CBZ) were purchased from Sigma. Topiramate (TOP) was purchased from TCI Europe. All compounds were dissolved in 100% DMSO and stored at −20°C.

### Microinjections Into Pericardium

Microinjections into the bloodstream via the common cardinal veins in pericardium at 3 dpf were performed as described previously (Van Dycke et al., 2019). In brief, 3 dpf zebrafish larvae were anesthetized by immersion in 0.4 mg/mL tricaine solution in Danieau's medium (Sigma-Aldrich, stock solution 4 mg/mL in Na_2_HPO_4_, pH 7–7.5), transferred to a 1.5% agarose mold imprint and positioned with the pericardium facing upwards. Injection glass capillaries (WPI, TW100F-4) were pulled with a micropipette puller (Sutter Instruments). Microinjections were performed using a M3301R Manual Micromanipulator (WPI) and a Femtojet 4i pressure microinjector (Eppendorf). An injection volume of 1 nl was applied. A 3 dpf zebrafish larva was determined to have an average weight of 0.25 mg.

### General Toxicity and Survival

VHC- or KA-injected (2.5, 5, 10 and 20 mg/kg) larvae were monitored under a stereomicroscope (Leica MZ10F microscope) from 1 day post injection (dpi) until 4 dpi for death, alterations in morphology and signs of toxicity (i.e., altered body axis/curvature, jaw malformations, uninflated swim bladder, yolk sac deformity, growth retardation and pericardial or eye edema). A larva was considered to respond normally upon touch stimulus if it swam away over a distance twice its body length. The severity of zebrafish larvae malformation was classified into four groups: “normal”—no visual malformation detected, “mild”—larvae have one malformation (most often the absence of swim bladder), “moderate”—<5 malformations and “severe”—five mild malformations or at least one severe abnormality, such as extreme edemas. The images were taken using Leica MZ10F microscope with a Leica DFC310 FX digital color camera and Leica Application Suite LASV 4.13 software.

### Estimation of KA and Neurotransmitters Levels by Liquid Chromatography With Tandem Mass Spectrometry (LC/MS)

KA and neurotransmitter content was determined using a previously described adapted method (Wojnicz et al., 2016). Five larval heads per sample from four independently injected batches were collected at 15 min post injection (mpi), 2 h post injection (hpi), 1 dpi, 2 dpi, 3 dpi and 4 dpi after injection of KA and their respective VHC-injected controls. As negative controls non-injected larvae of 3 dpf were used. Acidified water (ice-cold 1.89% formic acid in MilliQ water containing internal standard [d2-GABA, 10 mM]) was added to the samples, which were lysed by three cycles of freezing in liquid nitrogen by thawing and sonication for 60 s (pulses of 10 s on, 10 s off) at 150 W of intensity Qsonica 431C2 Cup Horn containing ice-cold water and attached to a Qsonica Q700 sonicator. Samples were centrifuged (20,000 g, 10 min at 4°C) and supernatants were transferred to new tubes to which ice-cold 1% formic acid in acetonitrile was added to precipitate proteins. Samples were vortexed, kept on ice for 10 min and centrifuged (20,000 g, 15 min at 4°C). Supernatants were transferred to new tubes and dried down under a stream of nitrogen. Samples were then reconstituted in ice-cold 5% acetonitrile, 0.2% formic acid in MilliQ water, transferred to HPLC vials and kept at 4°C before analysis. LC-MS/MS analysis was performed on a Sciex QTRAP 6500 mass spectrometer interfaced with a Shimadzu Nexera X2 HPLC system. Chromatographic separation was achieved using ACE C18-PFP 3 μm 4.6 × 150 mm HPLC column following ACE C18-PFP 3 μm 3.0 x 10 mm guard column with a gradient program at a flow rate of 0.2 mL/min at 40°C. The initial mobile phase conditions consisted of 95:5, v/v of 0.1% formic acid in MilliQ water and acetonitrile, which was maintained for 1.3 min. From 1.3 to 3.3 min, a linear gradient was applied up to a ratio of 10:90, which was maintained for 2.4 min. From 5.7 to 6.7 min, a linear gradient was applied down to the initial ratio of 95:5, then maintained for 2.6 min. Analytes were detected using MRM analysis in positive ion mode with the following mass transitions: KA 214.0 => 168.1, GABA 104.0 => 87.0, d2-GABA 106.0 => 89.0, glutamate: 148.0 => 84.0. Maximal signal (%) refers to the most intense signal found among measured samples per each analyte. Measured values were in the linear range of detection, as verified using standards.

### Behavioral Analysis

To evaluate locomotor behavior of VHC- and KA-injected larvae, an automated tracking device (ZebraBox, Viewpoint) was used. Zebrafish larvae were placed in a 96-well plate containing 100 μl embryo medium per well. Larval behavior was recorded for 150 min in alternating dark and light phases of 30 min, analyzed with Zebralab software (Viewpoint) and expressed as mean actinteg values (sum of all image pixel changes detected during 1 min interval) over 30 min time periods during 150 min of total recording.

To quantify seizure-like locomotor behavior, VHC- and KA-injected larvae were positioned in 1% methylcellulose. Images were acquired using a Leica DMi8 microscope at 10x magnification for 30 s with Leica LAS X software. Next, seizure-like tail twitching was defined as pixel changes for a specified part of the tail on a frame-by-frame basis and expressed as mean activity percentages throughout 30 s time interval using DanioScope Software (Noldus, Information Technology). Mean activity percentage of 100% implies that the gray scale of all pixels in the specified area was continuously changing throughout recorded time interval.

### Non-invasive Local Field Potential Recordings

Electrographic brain activity of VHC- or KA-injected larvae was assessed by non-invasive local field potential (LFP) recordings from the optic tectum as described previously (Siekierska et al., 2019). A glass electrode, connected to a high-impedance amplifier, was filled with artificial cerebrospinal fluid (124 mM NaCl, 2 mM KCl, 2 mM MgSO_4_, 2 mM CaCl_2_, 1.25 mM KH_2_PO_4_, 26 mM NaHCO_3_ and 10 mM glucose) and positioned on the skin above the optic tectum of a larva embedded in 2% low-melting point agarose (ThermoScientific). The differential signal between the recording electrode and the reference electrode was amplified 10,000 times by EXT-02F/2 extracellular amplifier (NPI Electronic), band pass filtered at 3–300 Hz and digitized at 2 kHz via a PCI-6251 interface (National Instruments) with WinEDR (John Dempster, University of Strathclyde). A HumBug noise eliminator (Quest Scientific) was used to remove 50–60 Hz noise. Each recording lasted for 10 min and was visualized with Clampfit 10.2 software (Molecular Devices Corporation). In order to quantify the signal from epileptiform brain discharges, we employed a specifically developed software (Hunyadi et al., 2017). In brief, LFP recordings were examined by Welch's power spectral density (PSD) analysis, using 100 ms long windows extracted with a Hamming window and 80% overlap. The window length was chosen considering that an epileptiform discharge may last as short as 50–100 ms. Subsequently, the average spectral power in consecutive 10 Hz frequency bands (i.e., 0–10 Hz, 10–20 Hz, …, 130–140 Hz) was computed. The magnitude of the average PSD values gives an indication about the amount of epileptiform discharges throughout the recordings in each frequency band.

### mRNA Extraction and qPCR

Total RNA from 10 heads of VHC- and KA-injected larvae at 3 hpi, 1 dpi and 2 dpi was extracted using TRIzol (Ambion, Life Technologies). Reverse transcription of total RNA to single-stranded cDNA was performed on 1 μg of total RNA using the High Capacity cDNA Reverse Transcription Kit (Applied Biosystems). Real-time PCR was performed in HardShell^®^ Low-Profile Thin-Wall 96-Well Skirted PCR Plates (Bio-Rad) using CFX96 Touch Real-Time PCR Detection System (Bio-Rad). Reaction mixture containing diluted cDNA template (1:20), primers specifically targeting genes of interest (Supplementary Table 1) and 2x SsoAdvanced Universal SYBR Green Supermix (Bio-Rad) was amplified under cycling conditions according to the manufacturer's protocol. Data generated were analyzed using CFX Manager Software (Bio-Rad). Transcripts were normalized against activating transcription factor 4 (*atf4*) housekeeping gene, experimentally determined to have the most stable expression in reaction conditions. The ΔΔCq method was used to determine the relative levels of mRNA expression between KA- and VHC-injected samples.

### Histology

VHC- and KA-injected larvae were fixed in 4% paraformaldehyde (PFA) at 4°C overnight and kept in 70% ethanol. At least four larvae per genotype group were embedded in 1% agarose in 1x TAE buffer. A specifically designed mold was used to produce agarose blocks with identically distributed wells of the same depth. Agarose blocks were gradually dehydrated in an enclosed automated tissue processor (Shandon Excelsior ES, Thermo Scientific) and subsequently embedded in paraffin. Heads of paraffin-embedded larvae were sectioned on a HM 325 manual rotary microtome (Thermo Fisher Scientific) at 5 μm thickness. Specimens were stained with haematoxylin and eosin (H&E) using Varistain™ Gemini ES Automated Slide Stainer (Thermo Fisher Scientific). Resulting sections were imaged at 20x and 40x magnification in SPOT 5.1 software (SPOT Imaging) by a SPOT-RT3 camera mounted on a Leica microscope. Brightness of the images was adjusted for the white background.

### *In vivo* Imaging of GABAergic and Glutamatergic Networks

VHC- and KA-injected *Tg[dlx5a/dlx6a-EGFP]* x *Tg[vglut2a:loxP-RFP-loxP-GFP]* zebrafish larvae were anesthetized with tricaine and immobilized in 2% low melting point agarose. For imaging of GABAergic and glutamatergic and networks in the optic tectum a Zeiss LSM 780—SP Mai Tai HP DS confocal microscope equipped with an LD LCI Plan Apo 25x/0.8 objective was used. The EGFP and RFP markers were excited at 488 and 561 nm, and emission recorded on Zeiss' spectral detector with bandpass windows set to 493–548 and 593–656 nm, respectively. Stacks were visualized and counted using Imaris 9.1.

### Pharmacological Evaluation

Prior to pharmacological assessment, maximum-tolerated concentrations (MTC) of the AEDs were determined as described previously (Li et al., 2020): VPA 50 μM, LEV 10 mM, TPM 100 μM, TGB 100 μM and CBZ 100 μM. KA-injected larvae at 2 dpi were individually incubated in 96 well-plates, with each well containing 100 μL of a freshly prepared AED solution at their MTCs. VHC- and KA-injected untreated larvae were incubated with 100 μL of control medium (1% DMSO). After 24 h, LFP recordings and quantification of seizure-like locomotor behavior were carried out as described above.

### Movies

Seizure-like locomotor behavior was captured in movies. For this, VHC- and KA-injected larvae injected were individually placed into a glass well (inner diameter: 7 mm, depth: 2 mm) filled with embryo medium and filmed for 20 s. The movies were made using a Carl Zeiss Stemi 2000-C stereomicroscope equipped with digital camera (Insight 2 Mp, Diagnostic Instruments) run by VisiView software (ID 1216) (Exposure time: 100 ms, time interval: 200 ms).

### Statistical Analysis

Statistical analyses were performed by GraphPad Prism 9. Unpaired, two-tailed *T*-test was used to compare means between two groups; for three or more groups one-way or two-way ANOVA followed by Bonferroni's or Sidak's multiple comparison test, respectively, was used. Data are presented as mean ± s.d. Zebrafish larvae were randomly allocated to experimental groups.

## Results

### KA-Injected Larvae Show Morphological Abnormalities

**Figure 1:**
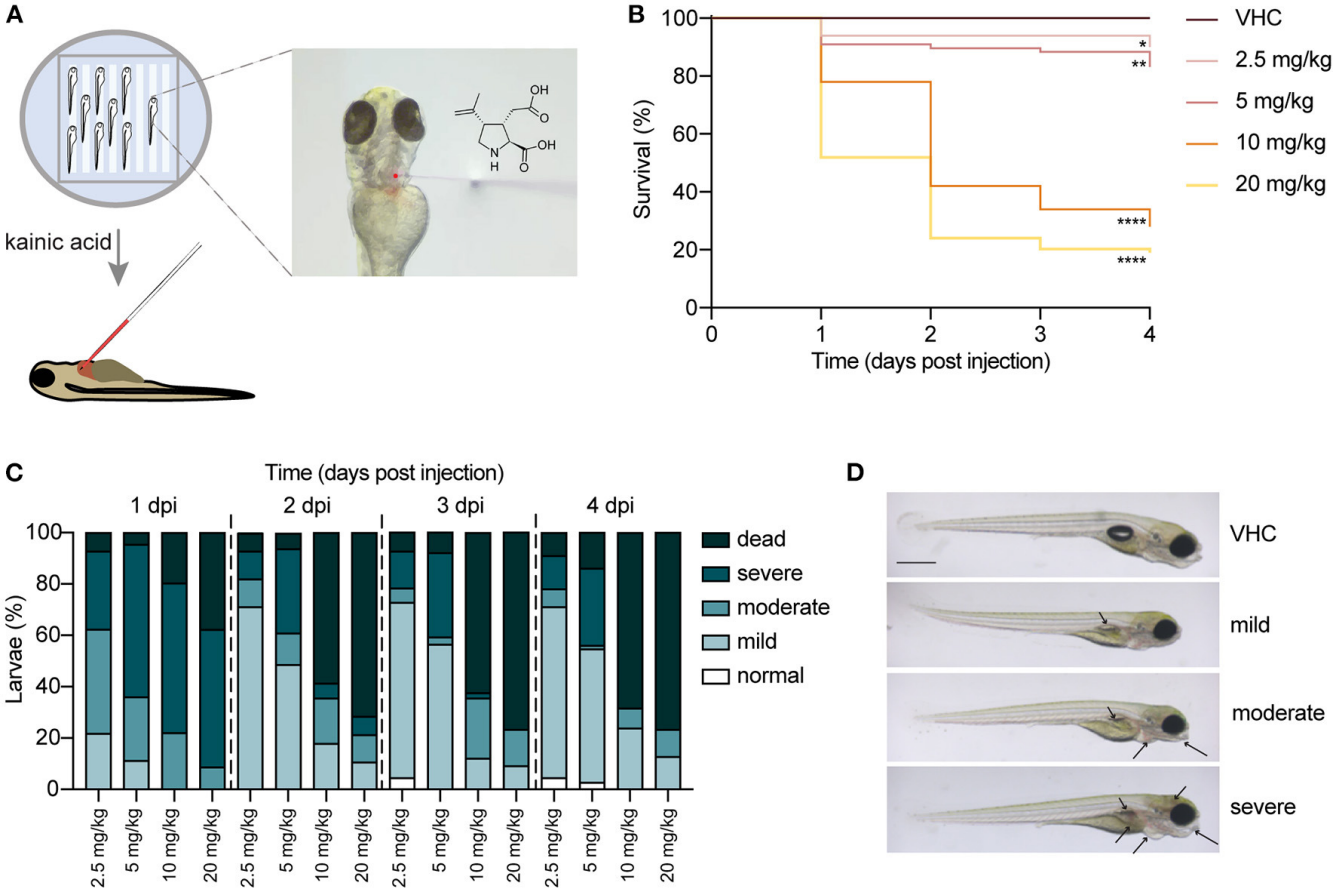
Injection of kainic acid into the bloodstream of 3 days post fertilization (dpf) zebrafish larvae. **(A)** A scheme of the set-up of kainic acid (KA) injection into 3 dpf zebrafish larvae. The larvae were anesthetized in 0.4 mg/mL tricaine and aligned in an agarose mold (left panel). KA mixed with dextran rhodamine B used as a tracer was injected into the zebrafish pericardium (right panel). **(B)** Survival of the larvae injected with 2.5, 5, 10, 20 mg/kg KA and vehicle (VHC) as a control. Significant larval death can be observed in a time-dependent manner. Statistical analysis between different doses injected was performed using Log-rank (Mantel-Cox) test (^*^p < 0.05, ^**^p < 0.01, ^****^p < 0.0001). Data pooled from three independent experiments, total numbers include: VHC *n* = 60, KA 2.5 mg/kg *n* = 50, KA 5 mg/kg *n* = 84, KA 10 mg/kg *n* = 61, KA 20 mg/kg *n* = 83. **(C)** Quantification of morphological abnormalities after injection of 2.5, 5, 10 and 20 mg/kg of KA from 1 until 4 days post injection (dpi). Larval phenotype was classified in function of number of malformations—normal, mild, moderate and severe. The graphs include the percentage of dead larvae. Data were pooled from three independent experiments, total numbers include: VHC *n* = 60, KA 2.5 mg/kg *n* = 50, KA 5 mg/kg *n* = 84, KA 10 mg/kg *n* = 61, KA 20 mg/kg *n* = 83. **(D)** Representative images of KA-injected larvae showing normal, mild, moderate and severe phenotypes. Mild or moderate abnormalities included predominantly lack of swim bladder, while severe abnormalities consisted of jaw malformation, pericardial edema and yolk sac edema. Arrows indicating morphological abnormalities (lack of swim bladder, jaw malformation, pericardial edema, yolk sac edema, opaque brain). Scale bar 500 μm.

In order to establish a new KA zebrafish model, we injected different doses of KA (2.5, 5, 10 and 20 mg/kg) into the pericardial cavity of 3 dpf *nacre* larvae (Figure 1A). First, their survival and health status were monitored for the subsequent 4 days post injection (dpi), until 7 dpf. As shown in Figure 1B, injection of 10 and 20 mg/kg of KA is toxic as it induces lethality in about 60 and 70% of the larvae respectively as early as at 2 dpi, in contrast to VHC-injected larvae. Conversely, more than 80% of the larvae injected with 2.5 and 5 mg/kg survived until 4 dpi (Figure 1B). Of note, 5 mg/kg injected larvae on average survived until 10 dpf (data not shown).

Additionally, we quantified morphological abnormalities caused by KA injection observed from 1 dpi to 4 dpi. Figure 1C illustrates that 2.5 and 5 mg/kg of KA induces mostly mild or moderate malformations. However, doses of 10 and 20 mg/kg KA caused overt toxicity signs in the survivors (Figure 1D). As a result, we used a single dose of 5 mg/kg of KA for all succeeding experimental work. This was the highest dose not inducing significant larval death, while causing only mild malformations in about 60% of injected larvae. Larvae with severe malformations were excluded from subsequent experiments.

### KA Injection Causes Acute Brain Inflammation and Cell Death

**Figure 2:**
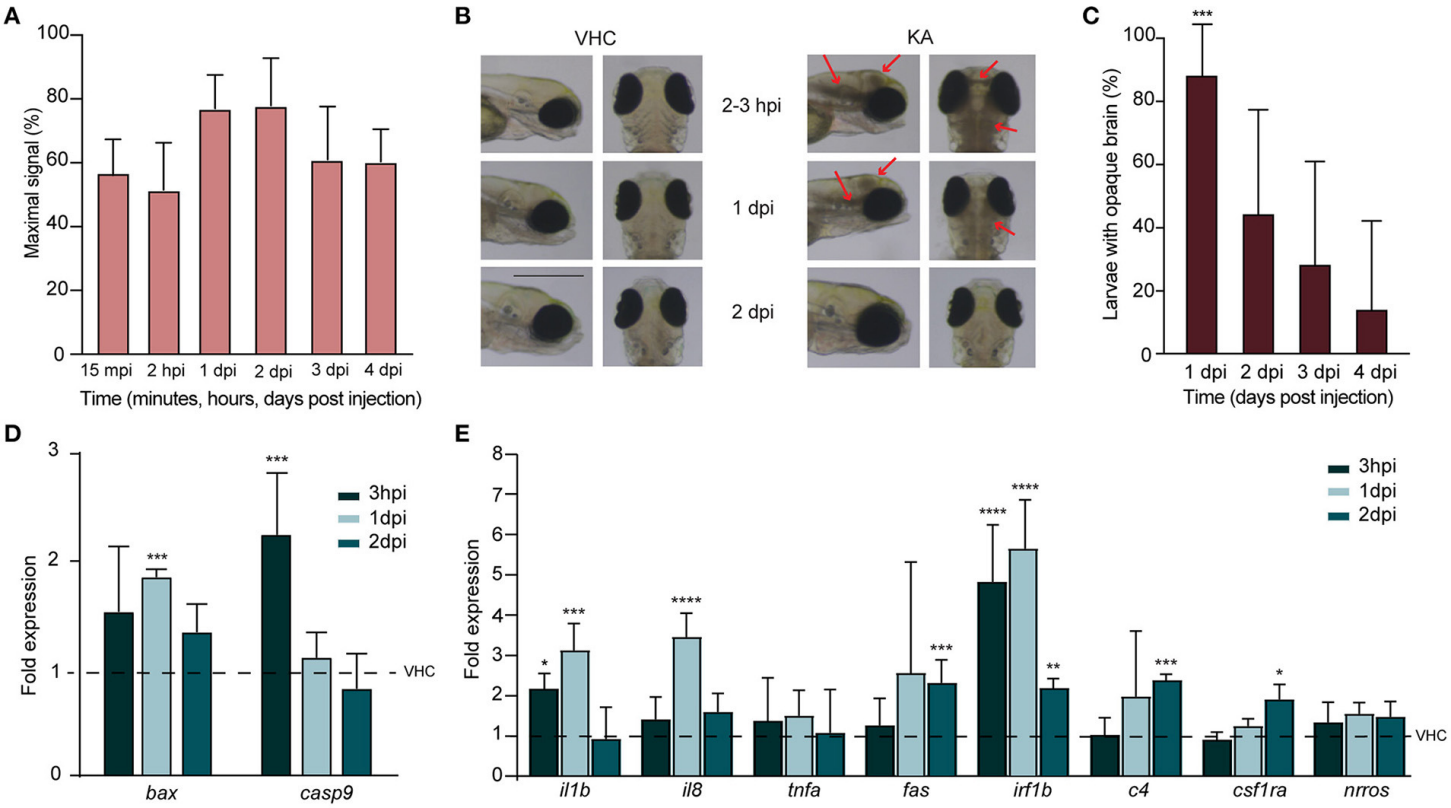
Injection of kainic acid in zebrafish larvae results in acute brain inflammation and apoptosis. **(A)** LC-MS/MS analysis of kainic acid (KA) levels in larval heads after injection of KA after 15 min post injection (mpi), 2 h post injection (hpi) and 1–4 days post injection (dpi) demonstrating stable levels of KA in zebrafish head tissues during each experimental timepoint. Maximal signal (%) refers to the highest signal found among measured samples. Data from four independent experiments are presented as mean ± s.d. **(B)** Representative images of VHC- and KA-injected larvae at 2–3 hpi upon 2 dpi. Characteristic opaque brain tissue was noticeable after 2–3 hpi and persisted mainly until 1 dpi. Red arrows mark the brain lesions. Scale bar 500 μm. **(C)** Quantification of larvae (%) with opaque brain at 1–4 dpi. All KA-injected larvae displayed opaque brain after 2–3 hpi (data not shown) and after 24 h still >80% of the larvae (^***^p < 0.001 compared to VHC determined immediately after injection [0%]) had this phenotype. Brain tissue cleared out during the following days. Means were pooled from three independent experiments, total number of larvae per condition: VHC *n* = 60, KA *n* = 84. Data are presented as mean ± s.d. Statistical analysis was performed using ordinary one-way Anova with Bonferroni's multiple comparison test. **(D,E)** Quantification of the expression levels of apoptosis **(D)** and inflammatory **(E)** markers in the heads of KA-injected larvae at 3 hpi, 1 dpi and 2 dpi by qPCR. Data were presented as fold expression to VHC-injected larvae at the respective timepoints (dotted line). Data from three independent experiments are presented as mean ± s.d. Statistical analysis was performed using ordinary one-way Anova with Bonferroni's multiple comparison test (^*^p < 0.05, ^**^p < 0.01, ^***^p < 0.001, ^****^p < 0.0001 compared to VHC set to a 1-fold expression).

First, we checked if KA was able to reach the brain of injected larvae and follow its dynamic at different timepoints post injection. LC-MS/MS analysis demonstrated that as soon as 15 mpi, KA reached the head and progressively accumulated to its highest experimental value at 1–2 dpi (77.0 ± 10.5% and 77.9 ± 14.8%, respectively) (Figure 2A). Somewhat unexpectedly, at 3 and 4 dpi KA was still detectable in the heads at comparable high levels (61.1 ± 16.6% and 60.4 ± 10.2%, respectively).

We found that KA injection at 5 mg/kg in 3 dpf larvae induced dense brain areas of opaque tissue in the head. This darkening of the brain was clearly observable and reached its maximum as early as after 2–3 hpi, then decreasing gradually afterwards and being cleared in the majority of the larvae by 2 dpi (Figures 2B,C). Because we hypothesized that such tissue opacity could originate from brain cell death, we analyzed the levels of pro-apoptotic markers by qPCR. We observed a significant upregulation of early apoptosis markers, *caspase-9* and *bax* at 3 hpi (2.3 ± 0.6 fold change) and 1 dpi (1.9 ± 0.1 fold change) respectively (Figure 2D). At 2 dpi no differences could be detected anymore. These results have been confirmed by acridine orange staining in KA-injected larvae demonstrating massive apoptosis occurring at 1 dpi, while being weakly detectable at 3 hpi and 2 dpi (Supplementary Figure 1). Of note, we also stained KA-injected larvae with propidium iodide, a vital dye labeling *in vivo* necrotic cells, however no evidence of necrosis was detected (data not shown).

We further investigated inflammatory response upon KA injection by measuring the expression levels of proinflammatory cytokines interleukin 1β (*il1b*), interleukin-8 (*il8*), and tumor necrosis factor alpha (*tnfa*) as well as *fas* and interferon regulatory factor 1β (*irf1b*) (Zou and Secombes, 2016; Campos-Sanchez and Esteban, 2021) in KA-injected larval heads. As early as 3 hpi, the levels of *il1b* and *irf1b* were significantly elevated (2.3 ± 0.4 fold change and 5.2 ± 1.5 fold change), and increased even more at 1 dpi together with *il8* being more than 3.3 ± 0.7, 3.7 ± 0.6 and 6 ± 1.3-fold higher in comparison to VHC-injected controls, respectively for *il1b, il8* and *irf1b* (Figure 2E). At 2 dpi *fas* was also significantly upregulated (2.5 ± 0.6 fold change). Additionally, we also examined the expression levels of *c4, csf1ra* and *nrros*, markers directly involved in inflammatory responses in human TLE (Mills et al., 2020), and two of them were significantly upregulated at 2 dpi (2.5 ± 0.2- and 2 ± 0.4 fold change respectively for *c4* and *csf1ra*, Figure 2E). Thus, we conclude that KA injections induced an acute inflammatory response in the brain associated to increased cell death.

### KA-Injected Larvae Display Whole Brain Abnormalities

**Figure 3:**
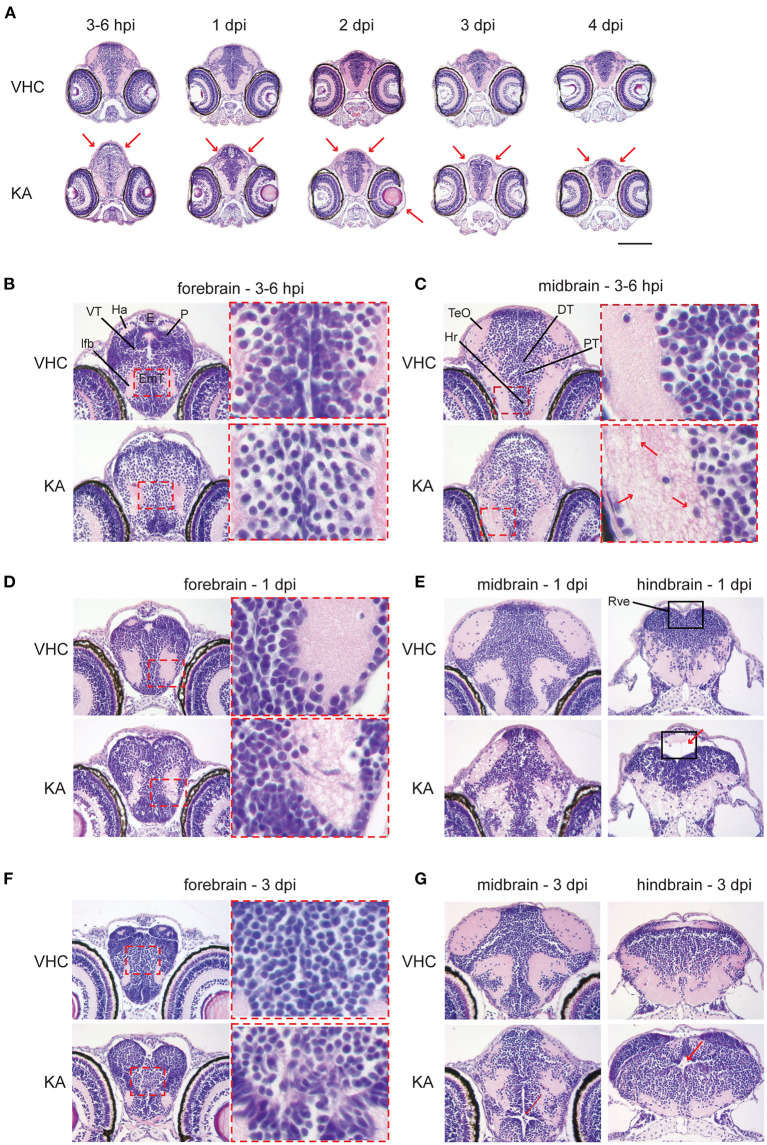
Histopathological analysis reveals that kainic acid (KA) injection induces whole brain abnormalities. Representative images of hematoxylin and eosin (H&E) staining on paraffin cross-brain sections of KA-injected larvae in comparison to VHC-injected siblings. **(A)** Abnormal brain shape was observed at 2–3 hpi until 4 dpi, marked with red arrows. Scale bar 500 μm. Selected brain abnormalities in KA-injected larvae from different parts of the brain at 3–6 hpi **(B,C)**, 1 dpi **(D,E)** and 3 dpi **(F,G)**. Red rectangles and arrows highlight the histopathological changes and its magnification. DT, dorsal thalamus; E, epiphysis; EmT, eminentia thalami; Ha, habenula; Hr, rostral hypothalamus; lfb, lateral forebrain bundle; P, pallium; PT, posterior tuberculum; Rve, rhombencephalic ventricle; TeO, optic tectum; VT, ventral thalamus.

Since we found that KA injection leads to an acute inflammation and to increased cell death in the brain, we further conducted histopathological analysis of brain sections of KA-injected larvae. Interestingly, this revealed major neuropathological changes upon systemic administration of KA (Figure 3A). Overall, KA-injected larvae had an oval-shaped brain and sporadically eye edema. At 3–6 hpi the brain of KA-injected larvae displayed obvious abnormalities throughout the whole brain such as isolated, less densely packed neuronal cell bodies (Figure 3B) and disruption of the white matter (eosin-positive regions) due to the presence of vacuolation (Figure 3C). The architecture of the brain was still disturbed at 1 dpi with disorganized arrangement of neuronal cell bodies in the gray matter, blurred delineation between gray and white matter, disrupted white matter and dilated ventricles (Figure 3D), especially the rhombencephalic ventricle (Figure 3E). The anomalous brain structures persisted until 2–4 dpi, most noticeable at the eminentia thalami region (Figures 3F,G), however the overall disruption of the brain organization was less pronounced suggesting a post-traumatic reorganization of brain tissues.

### KA-Injected Larvae Show Spontaneous Seizure-Like Behavior

**Figure 4:**
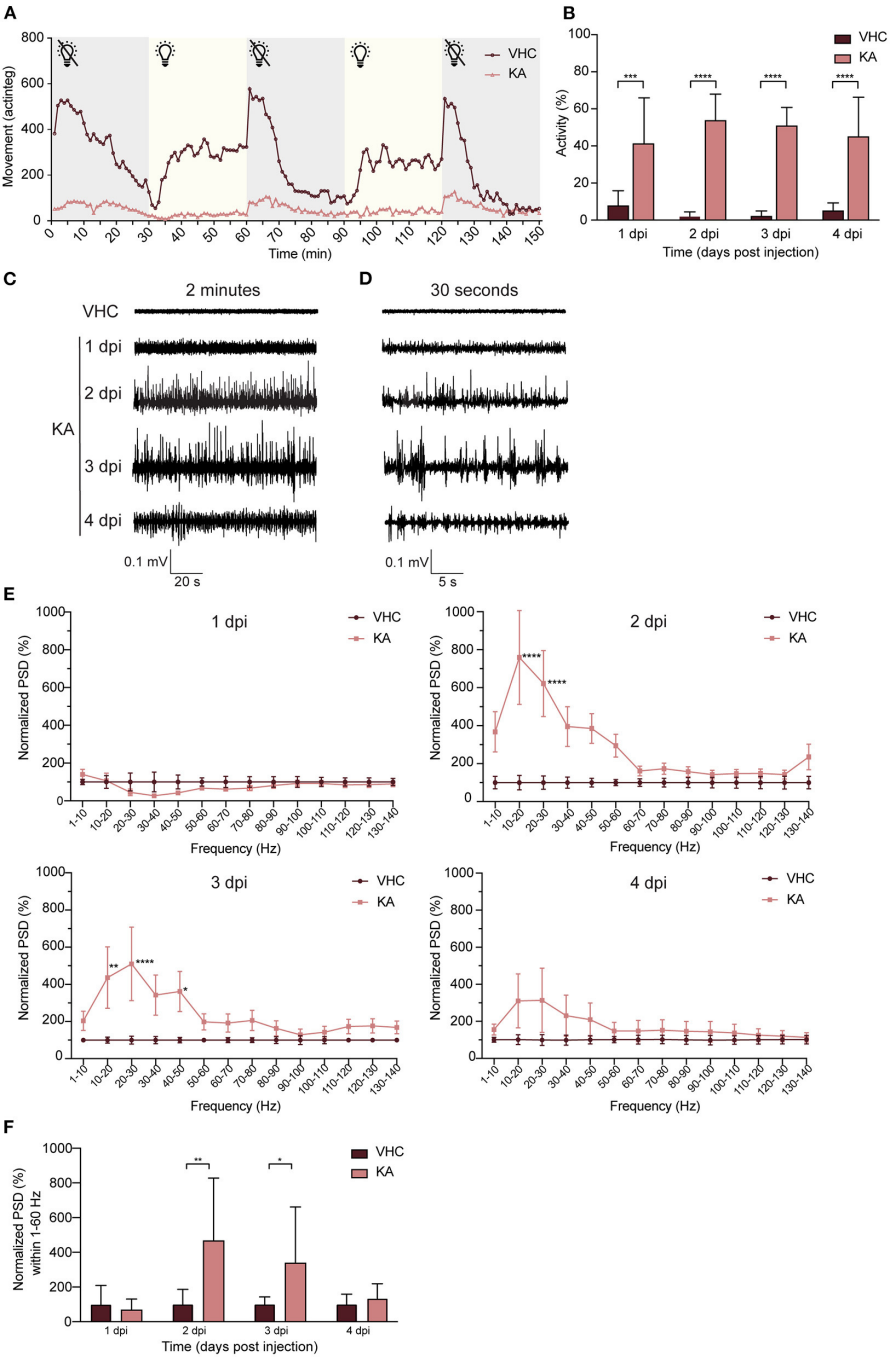
Kainic acid injected larvae display seizure-like behavior and continuous epileptiform discharges as measured by non-invasive local field potential recordings. **(A)** Decreased locomotor activity of KA-injected larvae at 3 dpi (*n* = 25) during interchanged dark and light periods of 30 min during 150 min in comparison to VHC-injected controls (*n* = 18). Error bars were not displayed for the sake of clarity. **(B)** Analysis of seizure-like behavior of KA-injected larvae at 1–4 dpi in comparison to VHC-injected age-matched controls by quantification of tail twitching. Total number of larvae per condition: 1 dpi VHC *n* = 10, KA *n* = 14; 2 dpi VHC *n* = 11, KA *n* = 11; 3 dpi VHC *n* = 14, KA *n* = 12; 4 dpi VHC *n* = 10, KA *n* = 14. At each time point KA-injected larvae displayed statistically significant increase in seizure-like behavior compared to VHC (^***^p < 0.001, ^****^p < 0.0001). Data are presented as mean ± s.d. Statistical analysis was performed using unpaired *t*-test. **(C)** Two-min representative local field potential (LFP) recordings from VHC- and KA-injected larvae at 1–4 dpi. Note the continuous epileptiform discharges in case of KA-injected larvae at 2–4 dpi. **(D)** Thirty-s magnification of the corresponding LFP recordings from **(C)**. **(E)** Power spectral density (PSD) analysis of KA and VHC-injected larvae at 1–4 dpi. No epileptiform brain activity could be observed in KA-injected larvae at 1 dpi, abnormal brain discharges appeared starting from 2 dpi. At 2 dpi and 3 dpi there was a statistically significant increase in PSD values in comparison to VHC-injected larvae. At 4 dpi, PSD values increased as well, however not significant. Results were normalized to VHC-injected larvae as 100%. Total number of larvae per condition: 1 dpi VHC *n* = 11, KA *n* = 10; 2 dpi VHC *n* = 10, KA *n* = 10; 3 dpi VHC *n* = 10, KA *n* = 10; 4 dpi VHC *n* = 9, KA *n* = 9. Statistical analysis was performed using two-way Anova with Sidak's multiple comparisons test (^*^p < 0.05, ^**^p < 0.01, ^****^p < 0.0001 compared to VHC). **(F)** Mean PSD values plotted per condition over the 1–60 Hz region for VHC- and KA-injected larvae. Statistically significant increases in mean PSD values compared to VHC were observed for 2 dpi (^**^p < 0.01) and 3 dpi (^*^p < 0.05). Data are presented as mean ± s.d. Statistical analysis was performed using unpaired *t*-test.

Immediately after KA injection, the larvae did not respond to any tactile stimulus to the body, in comparison to VHC-injected control larvae that showed a stereotypical startle and touch response. From 1 dpi onwards, KA-injected larvae displayed a weakened touch response and also demonstrated clear signs of locomotor impairment such as loss of posture and hypoactivity during both light and dark phases (Figure 4A). Concomitantly, at 1 dpi the KA-injected larvae exhibited stereotypical behavioral changes culminating in convulsion-like twitching that persisted repeatedly during the following days. Initially the larvae showed intermittent loss of posture, jerking, increased tail twitching and pectoral fin movement (Supplementary Movie 1). In some larvae this behavior developed into continuous tail and body waving (Supplementary Movie 2) culminating with vigorous whirlpool swimming and upwards darting (Supplementary Movie 3). In contrast, VHC-injected control larvae showed infrequent typical movement in small dart-like steps in all dimensions (Supplementary Movie 4). To quantify this seizure-like behavior, we recorded tail twitching movement using a high-resolution video camera capturing this subtle movement that could not otherwise be picked up using commercially available automated tracking systems (Supplementary Movies 5 [KA-injected], 6 [VHC-injected]). The data show that during 1–4 dpi, KA-injected larvae displayed significantly more tail twitching than age-matched VHC controls (5.3 ± 3.1, 25.6 ± 6.7, 23.4 ± 4.5 and 8.8 ± 4. fold change at 1–4 dpi respectively, Figure 4B).

### KA-Injected Larvae Show Spontaneous Epileptiform Discharges in the Brain

Non-invasive LFP recordings detect aberrant neuronal activity in zebrafish epilepsy and seizure models (Siekierska et al., 2019). Although no abnormal activity was detected at 1 dpi, KA-injected larvae typically showed continuous epileptiform brain discharges at 2–3 dpi, consisting of polyspiking discharges with amplitudes exceeding at least 3 times the baseline (Figures 4C,D). In contrast, recordings from VHC-treated larvae consistently showed baseline activity (Figures 4C,D). To quantify the results, we performed a PSD analysis by computing average power in consecutive 10 Hz frequency bands ranging from 1–140 Hz and normalized against VHC-treated larvae (Figure 4E). Epileptiform brain activity of KA-injected larvae at 1 dpi was not significantly different from VHC-treated larvae, whereas at 2–3 dpi there was a significantly higher PSD within the frequency range between 1 and 60 Hz (Figure 4E), confirming the frequency band characterizing polyspiking discharges (Schubert et al., 2014). At 4 dpi an increase was observed, however not statistically significant. Further, PSDs were plotted as mean PSD per condition over the 1–60 Hz region (Figure 4F) and in line with the linear representation at 2–3 dpi, there was a significant increase in the PSD values to 470.5 ± 221.9% and 342.0 ± 181.1% of VHC-injected controls respectively.

### KA-Injected Larvae Show a Decrease in GABAergic but Also Glutamatergic Neurons

**Figure 5:**
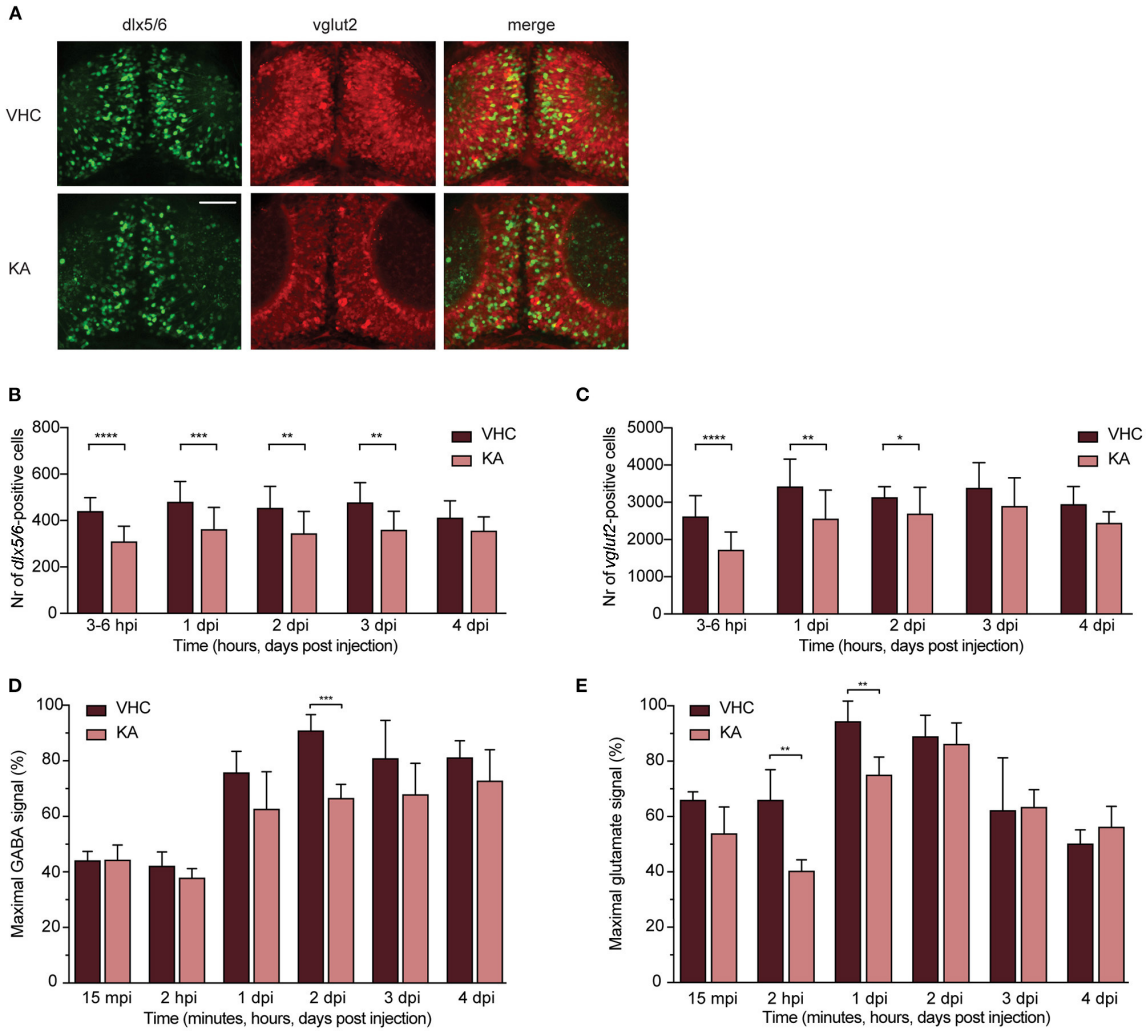
GABAergic and glutamatergic neuronal populations and neurotransmitters levels in kainic acid-injected larvae demonstrate a decrease. **(A)** Representative images of the GABAergic and glutamatergic neurons in transgenic reporter lines in 3–6 hpi VHC- and KA-injected larvae. Scale bar 50 μm. **(B)** Statistically significant decrease in the number of *dlx5/6*-positive cells was observed from 3–6 hpi until 3 dpi (^**^p < 0.01, ^***^p < 0.001, ^****^p < 0.0001). **(C)** Statistically significant decreases in the number of *vglut2*-positive cells were observed from 3–6 hpi until 2 dpi (^*^p < 0.05, ^**^p < 0.01, ^****^p < 0.0001). Data in **(B,C)** are presented as mean ± s.d. Total number of larvae per condition: 3–6 hpi VHC *n* = 20, KA *n* = 15; 1 dpi VHC *n* = 17, KA *n* = 16; 2 dpi VHC *n* = 16, KA *n* = 18; 3dpi VHC *n* = 13, KA *n* = 14; 4 dpi VHC *n* = 6, KA *n* = 6. Statistical analysis was performed using unpaired *t*-test. LC-MS/MS analysis of GABA **(D)** and glutamate **(E)** levels in the larval brains at 15 mpi, 2 hpi and 1–4 dpi. **(D)** Statistical reduction in GABA levels between VHC- and KA-injected larvae was observed at 2 dpi (^***^p < 0.001), **(E)** while in glutamate levels at 2 hpi (^**^p < 0.01) and 1 dpi (^**^p < 0.01). Maximal signal (%) in **(D,E)** refers to the highest signal found among measured samples per each neurotransmitter. Data from four independent experiments are presented as mean ± s.d. Statistical analysis was performed using unpaired *t*-test.

One of the key mechanisms underlying neuronal network hyperactivity is an imbalance of GABAergic (inhibitory) and glutamatergic (excitatory) transmissions in the brain (Brenet et al., 2019). To verify if such an imbalance is also present after KA injection in zebrafish larvae, we performed neuronal counting in double transgenic zebrafish larvae *Tg(dlx5a/dlx6a-EGFP)* x *Tg(vglut2a:loxP-RFP-loxP-GFP)*, expressing GFP-labeled GABAergic and RFP-labeled glutamatergic neurons (Figure 5A). The largest significant decrease in the number of *dlx5/6*-positive cells (GABAergic neurons) was observed as early as 3–6 hpi (from 439.9 ± 58.2 to 308.5 ± 66.1) and sustained until 3 dpi (from 480.5 ± 89.1 to 361.0 ± 94.8, from 453.6 ± 93.6 to 343.1 ± 95.0 and from 477.2 ± 85.9 to 358.1 ± 80.9 at 1–3 dpi respectively, Figure 5B). Likewise, a significant reduction in numbers of *vglut2*-positive cells (glutamatergic neurons) was seen as soon as 3–6 hpi (from 2613.0 ± 564.0 to 1718.0 ± 482.1) and sustained until 2 dpi (from 3417.0 ± 742.9 to 2551.0 ± 776.8 and from 3130.0 ± 287.2 to 2691.0 ± 710.9 at 1-2 dpi respectively, Figure 5C).

The observed decrease of both neuronal populations was further strengthened by titration of neurotransmitter levels. GABA levels largely dropped in KA-injected larvae specifically at 2 dpi (from 90.8 ± 5.9% to 66.5 ± 5.1%, Figure 5D). A similar trend was observed from 2 hpi until 4 dpi, however not statistically significant (Figure 5D). Glutamate levels were predominantly decreased between 3 and 6 hpi until 1 dpi, being statistically significant at 2 hpi and 1 dpi (from 65.0 ± 11.0 to 40.0 ± 4.1% and from 94.3 ± 7.5 to 75.0 ± 6.5% respectively, Figure 5E).

### Administering Anti-epileptics Alleviates Epileptiform Brain Activity but Not Seizure-Like Behavior in KA-Injected Larvae

**Figure 6:**
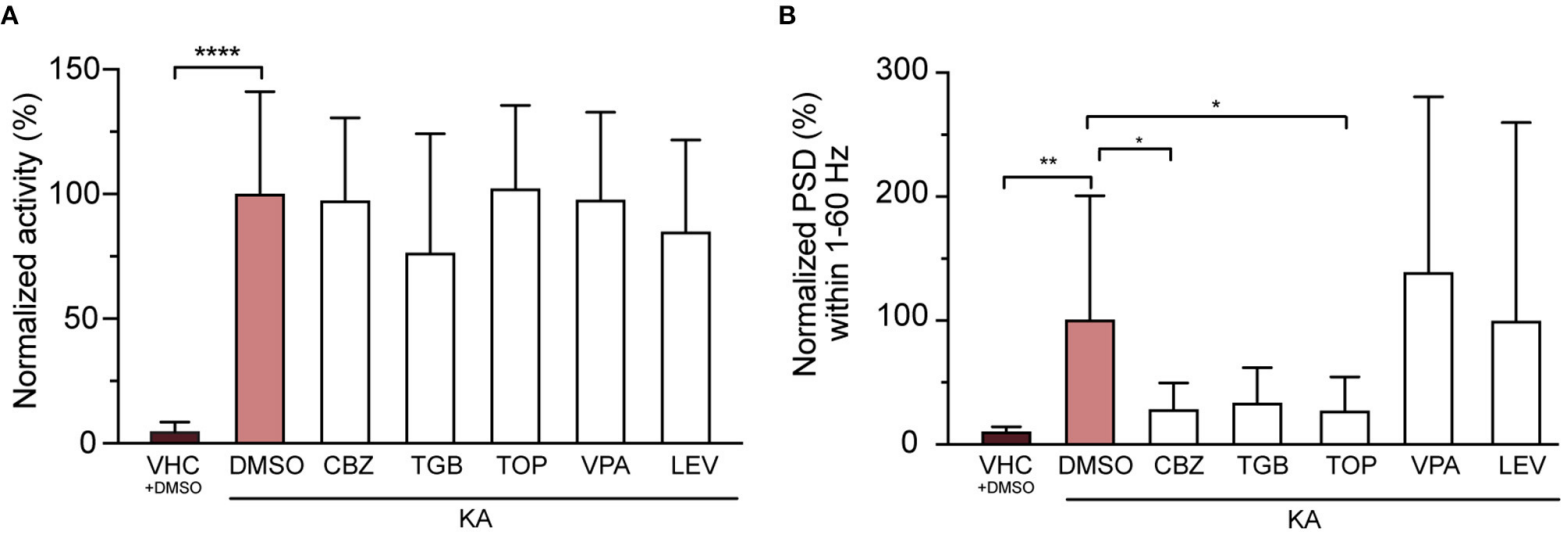
Three out of five tested anti-epileptic drugs (AEDs) rescue epileptiform brain discharges but not seizure-like behavior. **(A)** Quantification of seizure-like behavior at 3 dpi after incubation of VHC- and KA-injected larvae with commonly used AEDs or DMSO control for 24 h. None of the tested compounds decreased seizure-like behavior (tail twitching). Values were normalized to KA-injected controls treated with 1% DMSO as 100%. Total number of larvae per condition: VHC + DMSO *n* = 20, KA + DMSO *n* = 18, KA + CBZ *n* = 15, KA + TGB *n* = 15, KA + TOP *n* = 15, KA + VPA *n* = 15, KA + LEV *n* = 12. Data are presented as mean ± s.d. Statistical analysis was performed using ordinary one-way Anova with Bonferroni's multiple comparison test (^****^p < 0.0001). **(B)** Mean power spectral density (PSD) values from LFP recordings at 3 dpi plotted per condition over the 1–60 Hz region for VHC- and KA-injected larvae after incubation with AEDs or DMSO control for 24 h. Values were normalized to KA-injected controls treated with 1% DMSO as 100%. CBZ (^*^p = 0.04), TGB (ns, *p* = 0.05) and TOP (^*^p = 0.03) decreased epileptiform brain activity in KA-injected larvae (VHC ^**^p = 0.001), while VPA and LEV did not have any effect. Total number of larvae per condition: VHC + DMSO *n* = 18, KA + DMSO *n* = 38, KA + CBZ *n* = 15, KA + TGB *n* = 14, KA + TOP *n* = 14, KA + VPA *n* = 11, KA + LEV *n* = 12. Data are presented as mean ± s.d. Statistical analysis was performed using ordinary one-way Anova with Bonferroni's multiple comparison test. CBZ, carbamazepine; TGB, tiagabine; TOP, topiramate; VPA, valproic acid; LEV, levetiracetam.

We then tested anti-seizure effects of carbamazepine, tiagabine, valproic acid, topiramate and levetiracetam, five AEDs commonly used in epilepsy treatment (Androsova et al., 2017), on KA-injected larvae. The results showed that while none of the AEDs tested significantly affected the seizure-like behavior (tail twitching movement, Figure 6A), three out of five (CBZ, TGB and TOP) substantially reduced epileptiform brain events (Figure 6B), being significant for CBZ and TOP (reduction to 27.0 ± 21.7 and 26.5 ± 28.0%, respectively). Importantly, this indicates that both phenotypes (i.e., twitching and brain discharges) might not be caused by the same pathomechanisms.

## Discussion

Given the importance of gaining better insights into the pathogenesis of epilepsy, and the need for finding novel therapeutic strategies for this debilitating disease, the aim of this study was to generate a larval zebrafish-based KA epilepsy model. Such a validated model, recapitulating many characteristics of KA rodent models and hence reminiscent of the human TLE phenotype, could provide *in vivo* mechanistic insights into KA-induced seizures and processes preceding seizure onset.

Zebrafish larvae are established animals to study seizures and epilepsy (Baraban et al., 2013; Copmans et al., 2017; Yaksi et al., 2021) and an excellent model for medium-to-high throughput phenotypical drug screening as well as functional genomics studies at low costs (Patton et al., 2021). Zebrafish has an integrated nervous system as well as equivalent cellular and synaptic structure and function (Filippi et al., 2010; Panula et al., 2010). The brain of a zebrafish larva contains homologous structures to those found in mammals (Vaz et al., 2019) and already few days after fertilization, it can show a complex behavioral repertoire capable of sophisticated behaviors and susceptible to seizures (Stewart et al., 2012). Moreover, a recent review showed that zebrafish models of epilepsy are particularly reliable in terms of clinical relevance and pharmacological features (Griffin et al., 2018). Indeed, a clinical trial was recently launched for epilepsy (NCT04462770) with clemizole, a compound identified in zebrafish screen (Baraban et al., 2013).

As far as zebrafish seizure and epilepsy models are concerned, compounds typically used in chemically-induced models cause acute seizures, and not epilepsy (i.e., spontaneous recurrent seizures), hence no studies on epileptogenesis are feasible, and overall these models are considered to exhibit low construct validity. Conversely, genetic models are clinically relevant because they recapitulate the genetic defect present in patients, often mimicking the pathophysiological features of the human disease, including recurrent spontaneous seizures. Both genetic and chemical models have been successfully used for screening of novel anti-seizure compounds (Baraban et al., 2013; Copmans et al., 2019). Notably, our KA larval model combines characteristics of both model types, because the chemical insult eventually results in spontaneous recurrent seizures, thus epilepsy. In contrast to the well-established chemically-induced rodent chronic epilepsy models (Curia et al., 2008; Levesque and Avoli, 2013), a similar model was lacking in the zebrafish epilepsy field (Curia et al., 2008; Baraban et al., 2013; Levesque and Avoli, 2013; Copmans et al., 2018, 2019).

**Figure 7:**
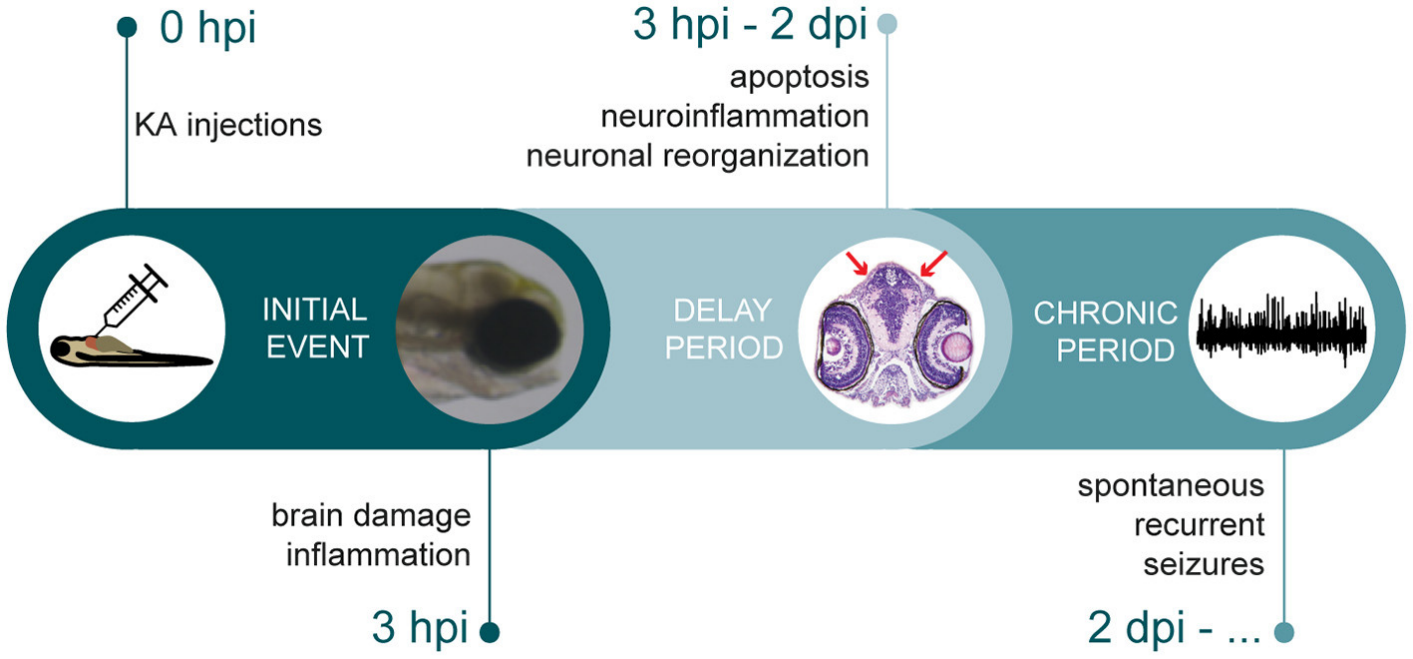
Timeline depicting multiple stages of epileptogenic processes observed in KA larval zebrafish model. After KA injection, immediate brain damage and acute inflammation could be observed at 3 hpi. This was followed by a delay period, where apoptosis, neuroinflammation and neuronal reorganization occurred. Finally, KA-injected larvae displayed spontaneous recurrent brain discharges.

KA together with its analog domoic acid are well-known to induce epilepsy in many different animals (Ramsdell and Gulland, 2014). For instance, sea lions exposed to domoic acid due to algal blooms develop hippocampal sclerosis and seizures (Cameron et al., 2019). Similarly, domoic acid induced seizure behavior, progressing from a brief hyperactive response into paralysis with tremors isolated in the tail, in 7 dpf zebrafish larvae via water immersion (Tiedeken and Ramsdell, 2009). However, best studied and characterized are the KA rodent models, where the phenotypes strongly resemble the clinical features observed in human TLE patients. KA rodent models are characterized by at least three different stages, starting off with an initial period of repetitive seizures (i.e., status epilepticus) that can be ultimately terminated by anticonvulsants. This stage is followed by a latency period lasting for 5 days up to one month, finally resulting in a chronic stage leading to a gradual development of SRSs (Pitkanen and Engel, 2014). In this study, direct delivery of KA into the bloodstream of zebrafish larvae resulted in a multiple stage process similar to the rodent model (Figure 7). Upon KA injection, immediate brain damage and acute inflammation could be observed, followed by a short delay period, where multiple events took place such as apoptosis, neuroinflammation and excitatory/inhibitory imbalance leading to abnormal brain reorganization. This ultimately resulted in sustainable spontaneous recurrent brain seizures. The remarkably short timeline during which KA rodent TLE pathology could be mimicked, underlines a major benefit of our zebrafish model making it an advantageous model to study epileptogenesis and chronic epilepsy.

Although the outcome of our study could be aligned with rodent models, differences could be discerned. After systemic injection of KA, generalized damage as revealed by the abundant presence of opaque tissue and histological analysis was observed in larval brains, in contrast to a more restricted damage in rodent brains. It is known that KA activates two types of receptors in the brain: kainate and AMPA receptors (Hampson and Manalo, 1998). In rodents neuronal loss is most prominent in regions with a high kainate receptor density: pyramidal cells of CA3, GABAergic interneurons of CA1/CA3, amygdala, basal ganglia, entorhinal cortex and cerebellum regions (Bahn et al., 1994; Lauri et al., 2005). In developing zebrafish larvae, kainate and AMPA receptors are expressed in the whole brain, spinal cord and retina (Ali et al., 2000; Hoppmann et al., 2008). Thus, a more generalized damage was seen in the larval brain most likely reflecting the widespread distribution of KA-responsive receptors.

Furthermore, in our KA zebrafish model a fast post-traumatic brain disorganization could be observed secondary to early brain damage (cell death and inflammation). Interestingly, we noticed a reduction of brain lesions and neuronal tissue defects at 3–4 dpi that could be explained by neural regeneration. The exact mechanisms of such a repair process are out of the scope of this work and are yet to be characterized. One can speculate that it might stem from the high capacity of fish for neurogenesis (Schmidt et al., 2013) due to acute local inflammation triggering regenerative responses (Kyritsis et al., 2012). Conversely, KA-triggered degeneration of the hippocampus in adult rodents is irreversible and spreads over time (Levesque and Avoli, 2013). Acute (neuro)inflammation and immune cell activation is among the first responses after a severe brain injury in both mammals and zebrafish (Kyritsis et al., 2012). A robust inflammatory response took place rapidly after KA injections as we observed a drastic increase in the expression levels of proinflammatory cytokines and inflammatory markers in KA-injected larval heads. This is in line with previously reported human TLE and KA rodent data (Mills et al., 2020; Ramazi et al., 2020). Importantly, it was shown previously that inflammatory mediators are implicated in the origin of seizures as well as in epilepsy progression (Vezzani et al., 2011). We hypothesize that an acute inflammatory reaction in our KA zebrafish model provides a context in which the molecular programs for regenerative neurogenesis could be initiated. Subsequently, neuronal cell death combined with increased neuroinflammation might have led to error-prone long-term brain tissue reorganization that resulted in reshaping of the brain circuitry prone to excitatory/inhibitory imbalance, thus epileptiform discharges in the brain. These assumptions open the door for more research to be conducted understanding the substratum of epileptogenesis for which our KA zebrafish model will be advantageous.

Epileptiform discharges were analyzed by their PSD to quantitatively demonstrate differences in specific frequency bands. Starting from only 2 dpi, in KA- and VHC-treated larvae major differences were observed in the 1–60 Hz range. Interestingly, comparable gamma oscillations occur in affected hippocampal areas of KA-treated rodents (Levesque and Avoli, 2013). Although we did not directly measure the electrographic discharges from the corresponding region due to technicalities of the non-invasive LFP technique as well as anatomical differences between zebrafish and rodent brain, we are convinced that the seizures observed are not a local phenomenon but reflect alterations in functional brain networks. This has been previously demonstrated by exposing zebrafish lines that express genetically-encoded calcium indicators to monitor neuronal activity to other proconvulsant chemicals, for instance PTZ (Turrini et al., 2017; Liu et al., 2019), where the epileptiform discharges clearly resembled the ones in our model, as well as other seizure and epilepsy zebrafish models (Baraban et al., 2005; Schubert et al., 2014; Griffin et al., 2018; Samarut et al., 2018; Gawel et al., 2020). Additionally, a similar but longer latency period has been previously reported in KA rodent models as well, where it takes five days to one month for seizures to first appear, although abnormalities in EEG patterns might occur beforehand (Levesque and Avoli, 2013).

Moreover, we show that although KA was still present at 3–4 dpi in the head of injected larvae, its damaging effects were limited to the first h post-injection, suggesting that KA might exhibit its harmful activity also acutely. In fact, a difference exists between the onset of locomotor abnormalities and seizure-like behavior on the one hand, and brain discharges on the other hand. We hypothesize that behavioral manifestations starting at 1 dpi originate from CNS areas that are not detected by the single electrode positioned at the optic tectum. This outcome further implies spatial-temporal changes in the neuronal network affecting different brain areas. However, we cannot exclude the possibility that the seizure-like behavior was caused by a continuous effect of KA, given the fact that it was present in the head of injected larvae during the experimental timeline.

Glutamatergic/GABAergic imbalance has been found as one of the key mechanisms underlying epilepsy. A transient decrease of GABAergic and glutamatergic neurons with a concomitant drop of the corresponding neurotransmitters was observed after KA injection. Although we did not report an obvious and sustained imbalance between both circuits, it leads us to speculate that the excitatory/inhibitory balance of normal brain function is affected. The results further show that the GABAergic and glutamatergic systems in the optic tectum recovered in 3–4 dpi period coinciding with lower epileptiform brain activity at 4 dpi. Hence, our model is also well-suited to investigate altered balances between glutamatergic excitation and GABAergic inhibition in the zebrafish neuronal networks.

Overall, the results show that after a dramatic excitotoxic KA-induced brain injury associated with neuro-inflammation, likely some unique brain repair mechanisms are launched in zebrafish larvae. Due to these post-traumatic rearrangements, the brain tissue is re-organized and re-wired into a dysfunctional neural circuitry that gets irreversibly damaged resulting in persistent seizure-like locomotor behavior and spontaneous epileptiform activity. We anticipate that the short period in which inflammatory, apoptotic and epileptogenic triggers as well as repair mechanisms are manifested, represents a great window of opportunity to study further the underlying biological processes. The outcome of our study can possibly support the future design of disease-modifying therapeutic strategies for the treatment of epileptic diseases. Moreover, as the KA-induced locomotor seizures were refractory to a set of clinically used AEDs, the KA zebrafish larval model could potentially be used as a pre-rodent model in the discovery of novel therapeutics in the fight against drug-resistant epilepsies.

## Data Availability Statement

The original contributions presented in the study are included in the article/Supplementary Material, further inquiries can be directed to the corresponding authors.

## Ethics Statement

The animal study was reviewed and approved by Ethics Committee of the University of Leuven (P061/2013, P023/2017, and P027/2019).

## Author Contributions

LH, D-HP, A-SD, ÉS, ASk, DC, YK, PV, and ASi performed the experiments and analyzed the data. LH, D-HP, PV, and ASi designed the experiments. PW conceived the study. PW and ASi supervised the study. LH, PW, and ASi wrote the manuscript. All authors contributed to the article and approved the submitted version.

## Funding

This work was supported by Fonds Wetenschappelijk Onderzoek [1S56820N to LH, 11F2919N to A-SD, G.0929.15 to PV] and KU Leuven [C32/18/067 to ASi, C32/17/044 to DC, METH/014/05 to PV].

## Conflict of Interest

ÉS is a co-founder of Modelis Inc. The remaining authors declare that the research was conducted in the absence of any commercial or financial relationships that could be construed as a potential conflict of interest.

## Publisher's Note

All claims expressed in this article are solely those of the authors and do not necessarily represent those of their affiliated organizations, or those of the publisher, the editors and the reviewers. Any product that may be evaluated in this article, or claim that may be made by its manufacturer, is not guaranteed or endorsed by the publisher.
