# Supplementary material for: Pericardial Injection of Kainic Acid Induces a Chronic Epileptic State in Larval Zebrafish
Source: Front Mol Neurosci. 2021 Oct 14;14:753936. doi: 10.3389/fnmol.2021.753936 (PMC8551382; doi:10.3389/fnmol.2021.753936)
Supplement: Supplementary file 1 [file Table_1.DOCX]

**Table S1**: Primers used for qPCR validation of inflammatory and apoptosis markers.

| **Gene** | **Forward primer sequence (5’ 🡪 3’)** | **Reverse primer sequence (5’ 🡪 3’)** |
| --- | --- | --- |
| *atf4* | TTAGCATTGCTCCGATAGC | GCTGCGGTTTTATTCTGCTC |
| *nrros* | AGCCGAAACAGGCTAACTGA | TTGGTGGGAAGTTCTGAAGG |
| *csf1ra* | ACTTTCCAGAACCCATGACG | CTCCGACGAAGAATCCAGAG |
| *c4* | ACAGTGAAGGGAGAGCTGGA | GCTCATGGGCTCATCATTTT |
| *fas* | GCAACGTCTGTTACCCCTGT | AGGATGAGTGGCACCAAAAC |
| *irf1b* | GTGGGTCAACAAGGAGGAGA | TGCTTGAACAGACAGGCATC |
| *bax* | GGCTATTTCAACCAGGGTTCC | TGCGAATCACCAATGCTGT |
| *casp9* | AAATACATAGCAAGGCAACC | CACAGGGAATCAAGAAAGG |
| *il1b* | GCTGGAGATCCAAACGGATA | ATACGCGGTGCTGATAAACC |
| *il8* | GTCGCTGCATTGAAACAGAA | AGGGGTCCAGACAGATCTCC |
| *tnfa* | GCGCTTTTCTGAATCCTACG | TGCCCAGTCTGTCTCCTTCT |
