# Supplementary material for: Pericardial Injection of Kainic Acid Induces a Chronic Epileptic State in Larval Zebrafish
Source: Front Mol Neurosci. 2021 Oct 14;14:753936. doi: 10.3389/fnmol.2021.753936 (PMC8551382; doi:10.3389/fnmol.2021.753936)
Supplement: Supplementary file 8 [file Data_Sheet_1.DOCX]

**SUPPLEMENTARY METHODOLOGY**

**Acridine orange and propidium iodide staining**

Acridine orange (AO) and propidium iodide (PI) used for live larvae staining were purchased from Sigma. AO and PI staining solutions were prepared at a concentration of 10 µg/ml in 1xE3 medium. Larvae were incubated at 28 °C for 15 minutes and afterwards carefully washed 3 times for 5 minutes in 1xE3 medium. Imaging was done using a Leica MZ10F fluorescent stereomicroscope, equipped with a Digital Color Camera Leica DFC310 FX (Sofware LAS 4.13). Filter set used for AO was GFP 10446222 and for PI - dsRED 10447079.
