# Supplementary figures and images for: Pericardial Injection of Kainic Acid Induces a Chronic Epileptic State in Larval Zebrafish
Source: Front Mol Neurosci. 2021 Oct 14;14:753936. doi: 10.3389/fnmol.2021.753936 (PMC8551382; doi:10.3389/fnmol.2021.753936)

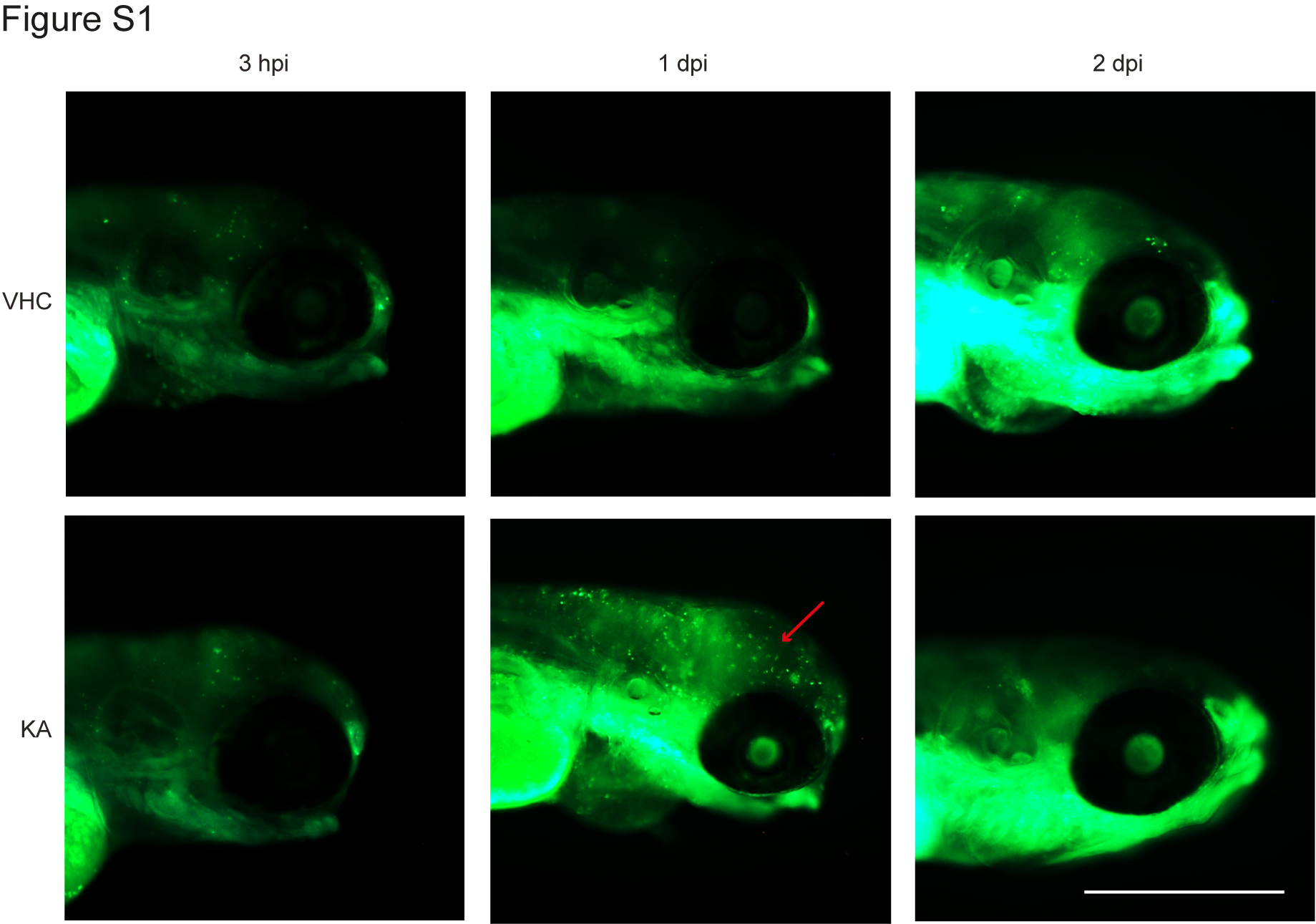

Supplement: Supplementary file 9 [file Image_1.TIF]
